# Multi-omics analysis reveals shape- and drying-dependent variation in non-volatile terpenoids of *Amomum tsao-ko* fruits

**DOI:** 10.3389/fpls.2026.1937993

**Published:** 2026-09-03

**Authors:** Mengli Ma, Lina Xiong, Hongbo Fu, Bingyue Lu

**Affiliations:** Yunnan Provincial Key Laboratory of Cross-Border Chinese Herbal Material, College of Agronomy, Honghe University, Mengzi, Yunnan, China

**Keywords:** *Amomum tsao-ko*, drying process, fruit morphotype, multi-omics integration, non-volatile terpenoids

## Abstract

*Amomum tsao-ko* is a valuable medicinal and culinary resource, yet its non-volatile terpenoid composition remains poorly characterized. This study integrated widely targeted metabolomics and transcriptomics to systematically profile non-volatile terpenoids in *A. tsao-ko* fruits and investigate the impacts of fruit morphotype and drying process. Using UPLC-MS/MS, 157 non-volatile terpenoids were identified, with 147 common across all samples, indicating a stable core metabolome. Fruit shape significantly influenced terpenoid accumulation, with round fruits generally exhibiting higher levels, and Caesalpin J identified as a shape-discriminatory marker. Drying induced profound alterations, promoting glycosylated forms while reducing free sesquiterpenoids and diterpenoids. Isosteviol was exclusively detected in dried fruits, serving as a drying-specific marker. Transcriptomic analysis revealed 49 shape-responsive differentially expressed genes enriched in terpenoid biosynthesis pathways. Weighted gene co-expression network analysis identified a terpenoid-associated module containing key structural genes and transcription factors. These findings provide a foundational chemical and molecular atlas for quality assessment, germplasm innovation, and optimized processing of *A. tsao-ko*.

## 1 Introduction

*Amomum tsao-ko* Crevost & Lemarié, a perennial herb belonging to the Zingiberaceae family, is a characteristic and economically significant plant endemic to southwestern China and adjacent regions of Southeast Asia (Lu et al., 2021). For centuries, its dried ripe fruits have held a dual status as a pivotal medicinal herb and an indispensable culinary spice, deeply embedded in the traditional practices and cultural heritage of local communities. In Traditional Chinese Medicine (TCM), *A. tsao-ko* is renowned for its properties of warming the middle jiao (spleen and stomach), dispelling cold, eliminating dampness, and resolving food stagnation. It is a key component in classical formulations such as the ‘Caoguo Yin’ decoction, used to treat ailments like malaria, epigastric pain, and digestive disorders (Fu et al., 2025). Concurrently, in the culinary realm, particularly in Yunnan, Sichuan, and Guizhou cuisines, *A. tsao-ko* is a fundamental spice prized for its unique penetrating aroma and ability to neutralize undesirable odors (e.g., from meat and fish), thereby enhancing the flavor profile of hot pots, stewed dishes, and various marinated foods (Ma et al., 2022). This convergence of medicinal and culinary value underscores its importance as a quintessential ‘medicine-food homology’ resource, driving substantial commercial demand in both the herbal medicine and food seasoning industries.

The pharmacological activities and distinctive flavor of *A. tsao-ko* are intrinsically linked to its complex profile of secondary metabolites. Existing phytochemical investigations have identified several classes of bioactive constituents, including essential oils (volatile terpenoids and phenylpropanoids) (Ma et al., 2024), diarylheptanoids, flavonoids (Fu et al., 2025), and phenolic acids (Fan et al., 2023). Among these, terpenoids constitute one of the most extensive and functionally diverse families of plant natural products. They play crucial roles in plant ecological interactions (e.g., defense against herbivores and pathogens, pollinator attraction) and serve as a rich reservoir for human therapeutics, exhibiting a broad spectrum of bioactivities such as anti-inflammatory, antimicrobial, antioxidant, and antitumor effects (Tholl, 2006). Terpenoids are biosynthesized via two primary, compartmentalized pathways: the mevalonate (MVA) pathway in the cytosol and the methylerythritol phosphate (MEP) pathway in plastids. Their structural diversity arises from the action of terpene synthases (TPSs), which generate the basic carbon skeletons, followed by modifications by cytochrome P450s, glycosyltransferases, and other tailoring enzymes (Ma et al., 2024).

Research on the terpenoid composition of *A. tsao-ko* has historically been skewed toward its volatile fraction. Gas chromatography-mass spectrometry (GC-MS) studies have characterized the essential oil, identifying dominant volatile monoterpenes and sesquiterpenes like 1,8-cineole, α-terpineol, and β-pinene, which are major contributors to its aromatic properties (Ma et al., 2024; Wen et al., 2025). In contrast, systematic investigation into its non-volatile terpenoid constituents, such as diterpenoids, triterpenoids, and their glycosylated derivatives remains markedly underdeveloped. These non-volatile compounds, often possessing higher molecular weights and greater structural complexity, are increasingly recognized for their potent and specific biological activities in other medicinal plants (Cai et al., 2021). For instance, in the closely related Zingiberaceae species like *Curcuma longa* and *Zingiber officinale*, non-volatile terpenoids like curcuminoids and gingerols are key bioactive principles (Paudel et al., 2025). A handful of non-volatile diterpenoids, including diterpenoids A–C, have been isolated from *A. tsao-ko* (Yang et al., 2022), hinting at a chemically rich but largely unexplored reservoir. A comprehensive, metabolomics-based profiling of these non-volatile terpenoids is therefore essential to fully understand the chemical basis of *A. tsao-ko*’s quality and efficacy.

Beyond mere compositional listing, understanding the factors that govern the accumulation and variation of these metabolites is critical for quality control and resource optimization. Two pivotal, yet understudied, factors in this context are fruit morphotype and post-harvest processing. In natural populations and cultivated accessions of *A. tsao-ko*, fruits exhibit noticeable phenotypic diversity. In medicinal plants, morphological variation is seldom neutral; it frequently correlates with underlying genetic diversity and differential expression of biosynthetic pathways, leading to distinct chemotypes (Chaudhary and Sharma, 2025). This phenomenon, where morphology serves as a visible marker for chemical composition, has been well-documented in species like *Panax ginseng* and *Houttuynia cordata* (Abaya et al., 2024; Zhang et al., 2025). However, in *A. tsao-ko*, the systematic impact of morphological diversity on the non-volatile terpenoid metabolome and the underlying molecular mechanisms responsible for potential differences remain unknown. Addressing this research gap is crucial for the scientific evaluation of germplasm resources, selective breeding for desirable chemotypes (with high yield or specific active compounds), and the establishment of objective, precise quality grading standards based on fruit morphology.

Furthermore, drying is an indispensable post-harvest processing step for *A. tsao-ko*, transforming the perishable fresh fruit into a stable, storable commodity (the dry fruit commonly used in TCM and cooking). This process is not merely dehydrative; it can induce profound chemical transformations through enzymatic and non-enzymatic reactions, including hydrolysis, oxidation, glycosylation, and Maillard reactions (Wen et al., 2025). Consequently, the metabolic profile of the dried product can differ significantly from that of its fresh counterpart. Such processing-induced alterations have been reported in various plants, where drying significantly modifies the profile of both volatile and non-volatile metabolites (Liang et al., 2025; Liu et al., 2023). For *A. tsao-ko*, the impact of drying has been briefly noted for volatile oils (Ma et al., 2024; Wen et al., 2025), but its effect on the non-volatile terpenoid landscape—arguably more relevant to its stored pharmacological potential and whether this effect is uniform or shape-dependent, represents a major research void. Elucidating these changes is fundamental for optimizing drying protocols to preserve or enhance desired bioactive components.

Therefore, to bridge the significant knowledge gaps concerning the non-volatile terpenoid metabolome of *A. tsao-ko*, its morphological and processing-induced variation, and its transcriptional regulation, we designed a comprehensive study integrating widely targeted metabolomics and transcriptomics. The specific objectives were: (1) to establish a UPLC-MS/MS-based method for the systematic profiling and characterization of non-volatile terpenoids in *A. tsao-ko* fruits; (2) to investigate the influence of different fruit shape and processing state on the accumulation patterns of these terpenoids, identifying shape-specific and drying-responsive marker metabolites; (3) to perform RNA-seq on different shapes fruits to uncover the transcriptional basis of shape-dependent terpenoid variation; and (4) to propose an integrated regulatory network linking transcriptomic changes to metabolomic outcomes. The findings from this study are expected to provide a foundational chemical and molecular atlas of non-volatile terpenoids in *A. tsao-ko*, offering scientific insights for its quality assessment, germplasm innovation, and optimal processing, ultimately supporting the sustainable and value-added development of this important medicinal and culinary resource.

## 2 Materials and methods

### 2.1 Plant materials

**Figure 1:**
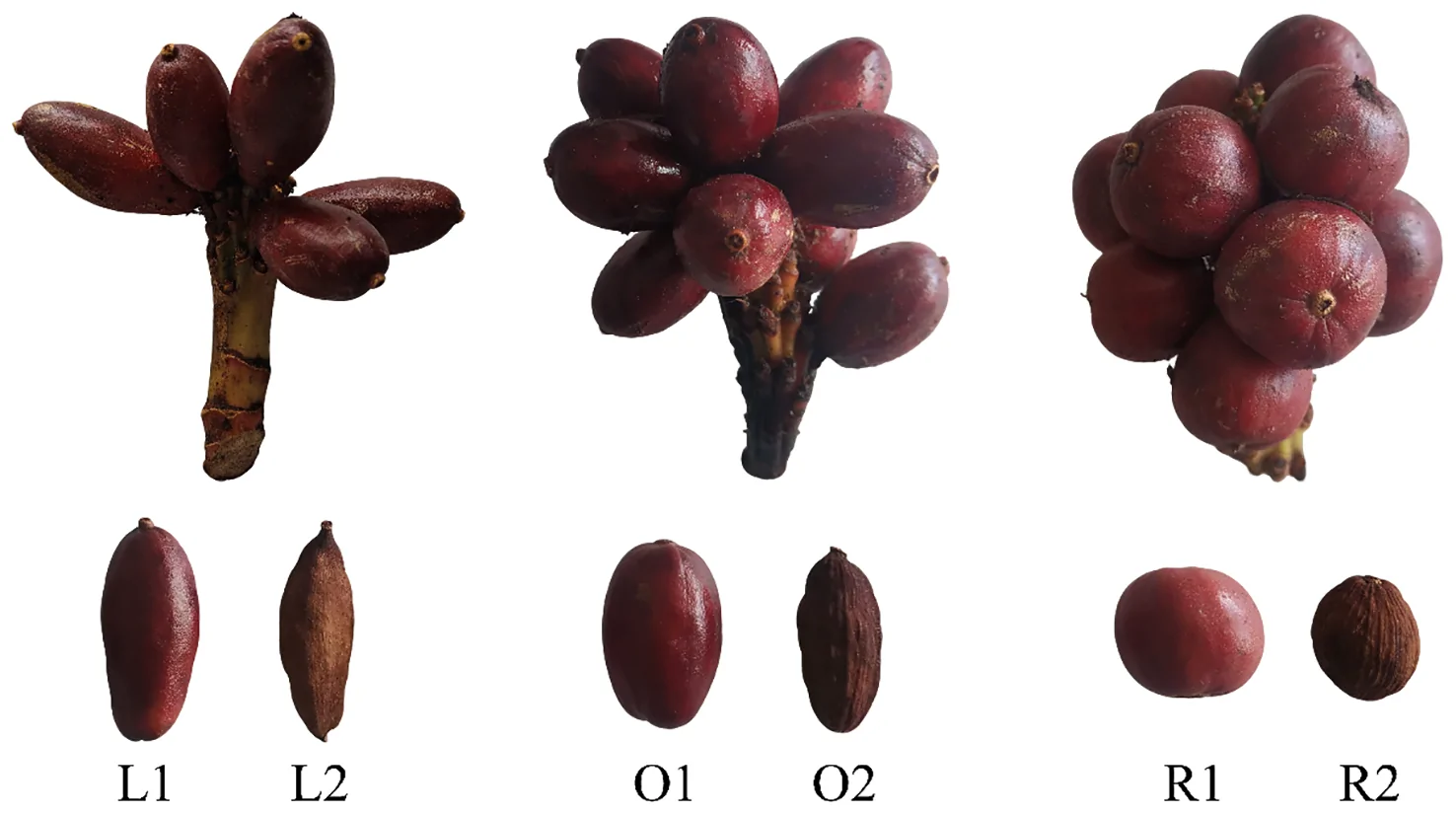
Phenotypic photographs of fresh and dried fruits of *Amomum tsao-ko* accessions with different shapes. L1, long fresh fruit; L2, long dried fruit; O1, oval fresh fruit; O2, oval dried fruit; R1, round fresh fruit; R2, round dried fruit.

Fresh and dried fruits from three distinct morphological types of *Amomum tsao-ko* accessions were used as experimental materials (**Figure 1**). These were classified based on length-width ratio (LWR) as long (LWR > 1.5), oval (1.5 > LWR > 1.1), and round (1.1 > LWR > 1.0) shapes (**Supplementary Figure 1**). Accordingly, the materials were designated as long fresh fruit (L1), long dried fruit (L2), oval fresh fruit (O1), oval dried fruit (O2), round fresh fruit (R1), and round dried fruit (R2). The ripened fruit was sampled on October 21, 2022, from Xinfazhai village, Dazhai Township County, Jinping Yao-Dai Autonomous County, Honghe Hani and Yi Autonomous Prefecture, Yunnan Province, China (22°88′78′′N, 103°33′E, 1,755 m). For each accession, three individual plants were selected, with each plant serving as a biological replicate, resulting in a total of three replicates. For each biological replicate (i.e., each individual plant), 20 fruits were peeled; subsequently, 10 peeled fruits were pooled into one sample, immediately frozen in liquid nitrogen, and processed in three biological replicates. The remaining 10 fruits were dried at 80 °C for 48 h, after which the peels were removed and combined into one sample, also with three biological replicates. Non-volatile terpenoid metabolites were analyzed in both fresh and dried fruit samples, while transcriptome sequencing was performed only on fresh fruits.

### 2.2 Analysis of non-volatile terpenoid metabolites

#### 2.2.1 Sample extraction

Biological samples were first freeze-dried using a vacuum freeze-dryer (Scientz-100F, Scientz, Ningbo, China), then ground into a fine powder with a grinder (mm MM 400, Retsch GmbH, Haan, Germany) at 30 Hz for 1.5 min. Precisely 50 mg of the resulting powder was weighed on an electronic balance (MS105D). Subsequently, 1200 µL of ice-cold (–20°C) 70% methanol-water extraction solvent (v/v) was added at a fixed ratio of 1200 µL per 50 mg sample. The mixture was vortexed for 30 seconds every 30 minutes, repeated six times in total. After centrifugation at 12000 rpm for 3 min, the supernatant was collected, passed through a 0.22 µm microporous membrane filter, and transferred into a vial for UPLC-MS/MS analysis.

#### 2.2.2 UPLC-MS/MS conditions

Sample extracts were analyzed using an ultra-performance liquid chromatography system coupled with an electrospray ionization tandem mass spectrometry system (UPLC-ESI-MS/MS). Chromatographic separation was performed on an Agilent SB-C18 column (1.8 µm, 2.1 mm × 100 mm) (Agilent Technologies, Santa Clara, CA, USA). The mobile phase consisted of solvent A (pure water with 0.1% formic acid) and solvent B (acetonitrile with 0.1% formic acid). The gradient elution program was set as follows: starting with 95% A and 5% B, followed by a linear increase to 95% B over 9 min, which was maintained for 1 min. The system was then returned to the initial conditions (95% A, 5% B) within 1.1 min and equilibrated for 2.9 min. The flow rate was 0.35 mL/min, the column temperature was set to 40 °C, and the injection volume was 2 µL.

Mass spectrometric detection was carried out using a triple quadrupole-linear ion trap mass spectrometer (QTRAP MS) operated in multiple reaction monitoring (MRM) mode. The ESI source parameters were as follows: ion source temperature, 500 °C; ion spray voltage, 5500 V (positive ion mode) and –4500 V (negative ion mode); ion source gas I, gas II, and curtain gas pressures were set to 50, 60, and 25 psi, respectively; collision gas pressure was set to high. Declustering potential and collision energy for each MRM transition were individually optimized. A specific set of MRM transitions was monitored for each analyte based on preliminary metabolite screening.

#### 2.2.3 Identification of non-volatile terpenoid metabolites

Qualitative identification was performed by matching the acquired mass spectra against the Metware database and utilizing secondary spectral information. Signals from isotopes, common adducts (e.g., K^+^, Na^+^, NH4^+^), and other high-molecular-weight fragments were excluded during data processing. For quantitative analysis, the MRM mode was employed. In this mode, precursor ions of target analytes were first selected in the first quadrupole (Q1), filtering out ions of other molecular weights to reduce interference. These precursor ions were then fragmented in the collision cell, and the resulting characteristic product ions were selectively filtered in the third quadrupole (Q3) for final detection. This approach enhances analytical specificity, accuracy, and repeatability. After processing all samples, chromatographic peaks were integrated, and the peaks corresponding to the same metabolite across different samples were aligned and normalized.

### 2.3 RNA sequencing

#### 2.3.1 Total RNA extraction, quantification, and qualification

Total RNA was extracted from plant tissues using the cetyltrimethylammonium bromide (CTAB) method combined with ethanol precipitation. Specifically, tissue samples (≥80 mg) were ground into a fine powder in liquid nitrogen and then transferred to 1.5 mL of CTAB extraction buffer. The mixture was incubated at 65 °C for 15 min in a thermal mixer to ensure complete inactivation of nucleases and denaturation of protein complexes. Subsequently, 400 μL of chloroform was added, thoroughly mixed for 5 min at 4 °C, and centrifuged at 12,000 × *g*. The aqueous phase was transferred to a new 2.0 mL tube, mixed with 700 μL of acidic phenol and 200 μL of chloroform, and centrifuged again at 12,000 × *g* for 10 min. Following another chloroform extraction of the aqueous phase, total RNA was precipitated by adding an equal volume of isopropanol and incubating at –20 °C for 2 h. After centrifugation at 12,000 × *g* for 20 min, the RNA pellet was washed once with 1 mL of 75% ethanol, air-dried in a biosafety cabinet, and finally dissolved in 50 µL of DEPC-treated water.

RNA quality and quantity were assessed using multiple instruments. Concentrations were measured with a Qubit^®^ 2.0 Fluorometer (Life Technologies, Carlsbad, CA, USA) and the Qubit™ RNA Assay Kit. Purity was verified using a NanoPhotometer^®^ spectrophotometer. RNA integrity and the absence of degradation or contamination were evaluated by 1% agarose gel electrophoresis and with an Agilent 2100 Bioanalyzer (Agilent Technologies, Santa Clara, CA, USA) using the RNA Nano 6000 Assay Kit.

#### 2.3.2 mRNA library construction

For each sample, 1 μg of total RNA was used as input for library preparation. Sequencing libraries were constructed following the manufacturer’s protocol for the NEBNext^®^ Ultra™ RNA Library Prep Kit (NEB). Briefly, mRNA was purified from total RNA using oligo(dT)-coupled magnetic beads and then fragmented. First-strand cDNA was synthesized using random hexamer primers and M-MuLV Reverse Transcriptase. Second-strand cDNA synthesis was subsequently performed using DNA Polymerase I and RNase H. The remaining overhangs were converted into blunt ends via exonuclease/polymerase activity. After adenylation of the 3’ ends, NEBNext adapters with hairpin loop structures were ligated to the cDNA fragments. To select cDNA fragments of preferentially 250–300 bp in length, the library fragments were purified using the AMPure XP system (Beckman Coulter).

Next, 3 μL of USER Enzyme (NEB) was used for adapter-ligated cDNA at 37°C for 15 min, followed by 5 min at 95°C. PCR amplification was then performed with Phusion High-Fidelity DNA polymerase, universal PCR primers, and index (X) primers. Finally, the PCR products were purified, and library quality was assessed using the Agilent Bioanalyzer 2100 system.

#### 2.3.3 Sequencing

Following the manufacturer’s specifications, the index-coded samples were clustered on a cBot Cluster Generation System using the TruSeq PE Cluster Kit v3-cBot-HS (Illumina, San Diego, CA, USA). After cluster generation, the prepared libraries were sequenced on an Illumina platform to generate 150 bp paired-end reads.

### 2.4 Data analysis

Data analysis was conducted using Microsoft Office 2021 (Microsoft Corporation, Redmond, WA, USA). Figures were generated with Origin 2021 (OriginLab Corporation, Northampton, MA, USA). Differential non-volatile terpenoid metabolites (DNVTMs) were identified based on a variable importance in projection (VIP) score > 1 from orthogonal projections to latent structures discriminant analysis (OPLS-DA) and an absolute log_2_ fold-change (|log_2_FC|) ≥ 1.0. The OPLS-DA model (**Supplementary Table 1**), including its score plot and loading plot, was constructed using the MetaboAnalystR R package. Prior to OPLS-DA, data were log_2_-transformed and mean-centered. A permutation test (200 iterations) was performed to validate the model and prevent overfitting.

For transcriptomic data, differentially expressed genes (DEGs) between comparison groups were identified using the DESeq2 package. The resulting P-values were adjusted using the Benjamini-Hochberg method to control the false discovery rate. Genes with an adjusted P-value < 0.05 and |log_2_FC| ≥ 1 were considered significantly differentially expressed.

An integrative multi-omics correlation network was constructed to explore relationships between key DNVTMs and DEGs. Pairwise Pearson correlation coefficients were calculated, and associations with a correlation coefficient > 0.8 and a P-value < 0.01 were selected. The network was visualized using Cytoscape 3.9.1. Furthermore, Weighted Gene Co-expression Network Analysis (WGCNA) was performed using the blockwiseModules function with the following parameters: minimum module size = 50 and merge cut height = 0.25 (other parameters set as default). The correlation between the module eigengene (ME) of each co-expression module and the levels of key non-volatile terpenoids was calculated to identify terpenoid-associated specific modules.

Various visualizations were generated using R and Python: Venn diagrams and Principal Component Analysis (PCA) plots were created in R (version 3.5.1). Sankey diagrams and advanced heatmaps were produced using R (version 4.2.0). Enrichment bubble plots were constructed with Python (version 3.6.6).

## 3 Results

### 3.1 Comprehensive profiling and characteristic analysis of non-volatile terpenoids

**Figure 2:**
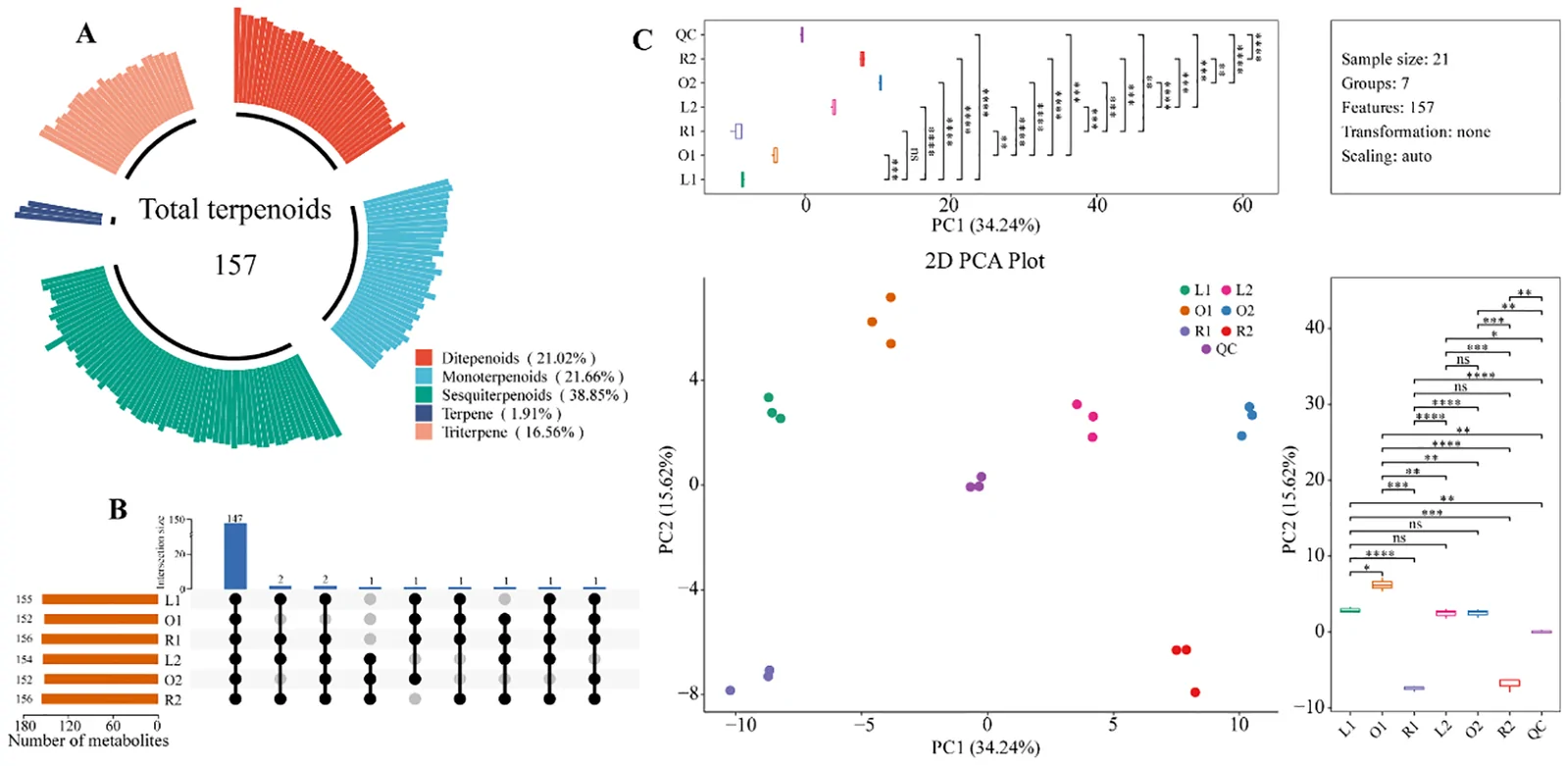
Comprehensive analysis of non-volatile terpenoids in fresh and dried fruits of *Amomum tsao-ko* with different morphotypes. **(A)** Classification of the identified non-volatile terpenoid metabolites. **(B)** Abundance, overlap, and specificity of the identified non-volatile terpenoid metabolites across different samples. **(C)** Principal component analysis and statistical validation based on the identified non-volatile terpenoid metabolites.

A comprehensive widely targeted analysis of non-volatile terpenoids in *Amomum tsao-ko* fruits was performed using UPLC-MS/MS. A total of 157 terpenoid metabolites were identified and classified (**Supplementary Table 2**; **Figure 2A**). These included 34 monoterpenoids, 61 sesquiterpenoids, 3 terpenes, 33 diterpenoids, and 26 triterpenoids, establishing a detailed chemical profile for the investigated samples.

Analysis of terpenoid distribution across the six sample groups, including fresh and dried fruits of long shapes (L1, L2), round shapes (R1, R2), and oval shapes (O1, O2) shapes revealed a high degree of commonality. The number of terpenoids detected per sample was highly consistent: L1 (155), R1 (156), O1 (152), L2 (154), R2 (156), and O2 (152) (**Figure 2B**). UpSet analysis confirmed that 147 terpenoids (93.63% of the total) were common to all six sample groups. No sample-unique terpenoids were identified. Notably, three metabolites served as potential characteristic markers: Isosteviol (a diterpenoid) was exclusively detected in all dried fruit samples (L2, R2, O2) but absent in fresh fruits, identifying it as a candidate marker for the dried state. 8-hydroxy-1,4a-dimethyl-7-(prop-1-en-2-yl)-4,4a,5,6,7,8-hexahydronaphthalen-2(3H)-one and Curcumenol (both sesquiterpenoids) were consistently present in both fresh and dried fruits of long shapes (L1, L2) and round shapes (R1, R2) shapes but were absent in all oval-shaped samples (O1, O2). These two metabolites are therefore proposed as shape-specific markers distinguishing long/round from oval fruits.

Principal component analysis (PCA) was conducted based on the abundance profiles of all 157 terpenoids to visualize global metabolic differences. The first two principal components (PCs) explained 49.86% of the total variance (PC1: 34.24%, PC2: 15.62%) (**Figure 2C**). The PCA score plot revealed a clear separation of samples primarily along PC1. Notably, all fresh fruit samples (L1, O1, R1) clustered with negative PC1 scores, whereas all dried fruit samples (L2, O2, R2) clustered with positive PC1 scores, indicating that PC1 predominantly captures the variance associated with the drying process. Furthermore, fresh samples showed a tendency to separate according to fruit shape along PC2.

To statistically evaluate the observed separations, pairwise Student’s t-tests were performed on the PC1 and PC2 scores. PC1 primarily distinguished samples based on processing state. Highly significant differences (*p* < 0.01) were consistently observed between all fresh (L1, O1, R1) and dried (L2, O2, R2) sample pairs, irrespective of their morphotype (e.g., L1 vs. L2, *p* = 4.4e-06; O1 vs. O2, *p* = 1.9e-05; R1 vs. R2, *p* = 0.00017). In contrast, no significant difference was found between fresh long and round shapes fruits (L1 vs. R1, *p* = 0.301). This confirms that the drying process induces the most substantial alteration in the non-volatile terpenoid profile. PC2 captured variance related to fruit morphotype, with a notable interaction with the processing state. The pattern was more complex. In the fresh state, all three morphotypes showed significant separation from each other on PC2 (L1 vs. R1, *p* = 6.5e-06; L1 vs. O1, *p* = 0.011; O1 vs. R1, *p* = 0.00026). After drying, the distinction between long and oval shapes fruits diminished (L2 vs. O2, *p* = 0.996), while round shapes fruits remained significantly different from both long (L2 vs. R2, *p* = 0.0003) and oval (O2 vs. R2, *p* = 0.00039) shapes fruits. Notably, for a given morphotype, the fresh and dried samples did not differ significantly on PC2 (L1 vs. L2, *p* = 0.449; R1 vs. R2, *p* = 0.409), suggesting that the morphotype-associated metabolic signature is largely preserved during drying. Quality control (QC) samples clustered tightly near the origin of the PCA plot, demonstrating the high reproducibility and stability of the analytical platform throughout the sequence.

### 3.2 Analysis of highly abundant non-volatile terpenoids and their distribution patterns

**Figure 3:**
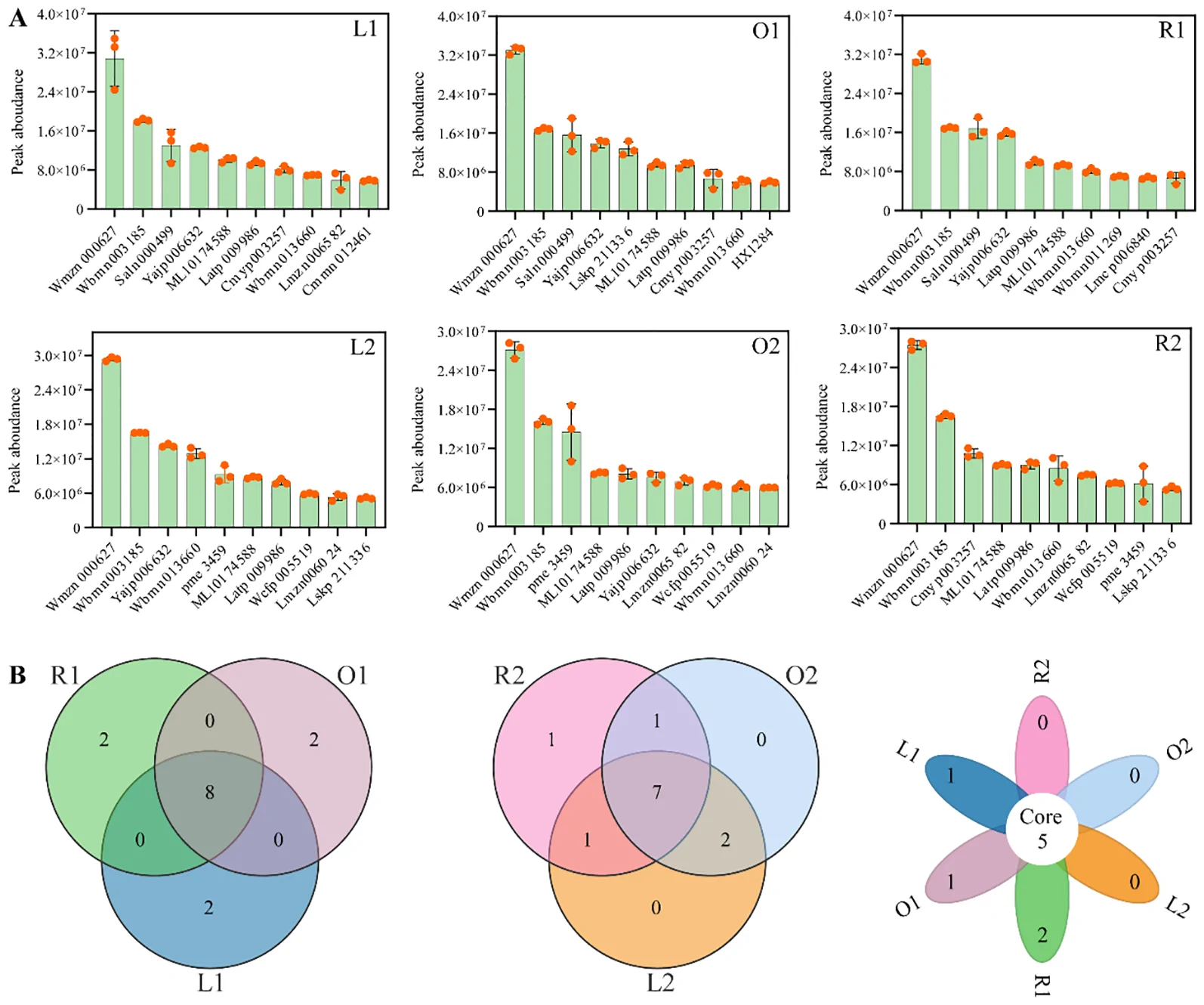
Analysis of highly abundant non-volatile terpenoids. **(A)** Abundance of the top ten most abundant non-volatile terpenoid metabolites in each sample. **(B)** Venn diagram illustrating the overlapping and unique high-abundance non-volatile metabolites across different samples.

To identify the core terpenoid constituents, the top 10 most abundant non-volatile terpenoid metabolites in each of the six samples were analyzed and visualized (**Figure 3A**). The diterpenoid Ajugalaevigatic acid and the sesquiterpenoid Kobusone were consistently the two most abundant metabolites across all samples, highlighting their role as fundamental chemical components of *Amomum tsao-ko* regardless of morphotype or processing state.

Venn analysis (**Figure 3B**) of the top-abundant metabolites revealed distinct shared and specific patterns. Among fresh fruits (L1, O1, R1): Eight metabolites were common high-abundance constituents, including Icariside B1, 7-O-(4’’-O-glucosyl)coumaroyl-loganic acid, (4R,7aS)-4-methyl-1,3,4,7a-tetrahydrocyclopenta[c]pyran-7-carbaldehyde, Confertifoline, Kobusone, 3,9-Dihydroxy-13(14)-labden-16,15-olide, Ajugalaevigatic acid, and 6β-Hydroxy-8α-methoxyeremophila-1(10),7(11)-dien-12,8β-olide. Morphotype-specific markers among the top 10 metabolites were also identified: Dehydroabietic acid and Colubrinic acid were unique, high-abundance constituents in L1, Caesalpin J and Dehydrololiolide in O1, and Vitexilactone and Spathulenol in R1.

Among dried fruits (L2, O2, R2): Seven metabolites were shared in high abundance: Confertifoline, Cafestol, Kobusone, 3,9-Dihydroxy-13(14)-labden-16,15-olide, 5-hydroxyindene-1,3-dione, Ajugalaevigatic acid, and 6β-Hydroxy-8α-methoxyeremophila-1(10),7(11)-dien-12,8β-olide. Notably, no metabolite was uniquely abundant in only L2 or O2. However, Icariside B1 was found as a high-abundance metabolite exclusive to dried round fruits (R2).

An intersection of all six sample sets identified five metabolites that constitute a stable “core” high-abundance terpenoid profile: Confertifoline, Kobusone, 3,9-Dihydroxy-13(14)-labden-16,15-olide, Ajugalaevigatic acid, and 6β-Hydroxy-8α-methoxyeremophila-1(10),7(11)-dien-12,8β-olide. The monoterpenoid 7-O-(4’’-O-glucosyl)coumaroyl-loganic acid was a characteristic high-abundance component shared specifically among fresh fruits, whereas the diterpenoid Cafestol and the sesquiterpenoid 5-hydroxyindene-1,3-dione were commonly abundant markers for dried fruits.

This analysis delineates a hierarchy of terpenoid abundance: a universal core, constituents specific to processing state (fresh vs. dried), and others associated with particular fruit morphologies, providing a quantitative foundation for the differential patterns observed in the multivariate analysis.

### 3.3 Comprehensive analysis of differential non-volatile terpenoid metabolites in *Amomum tsao-ko* fruits

#### 3.3.1 Differential non-volatile terpenoids among fresh fruits of different shapes

**Figure 4:**
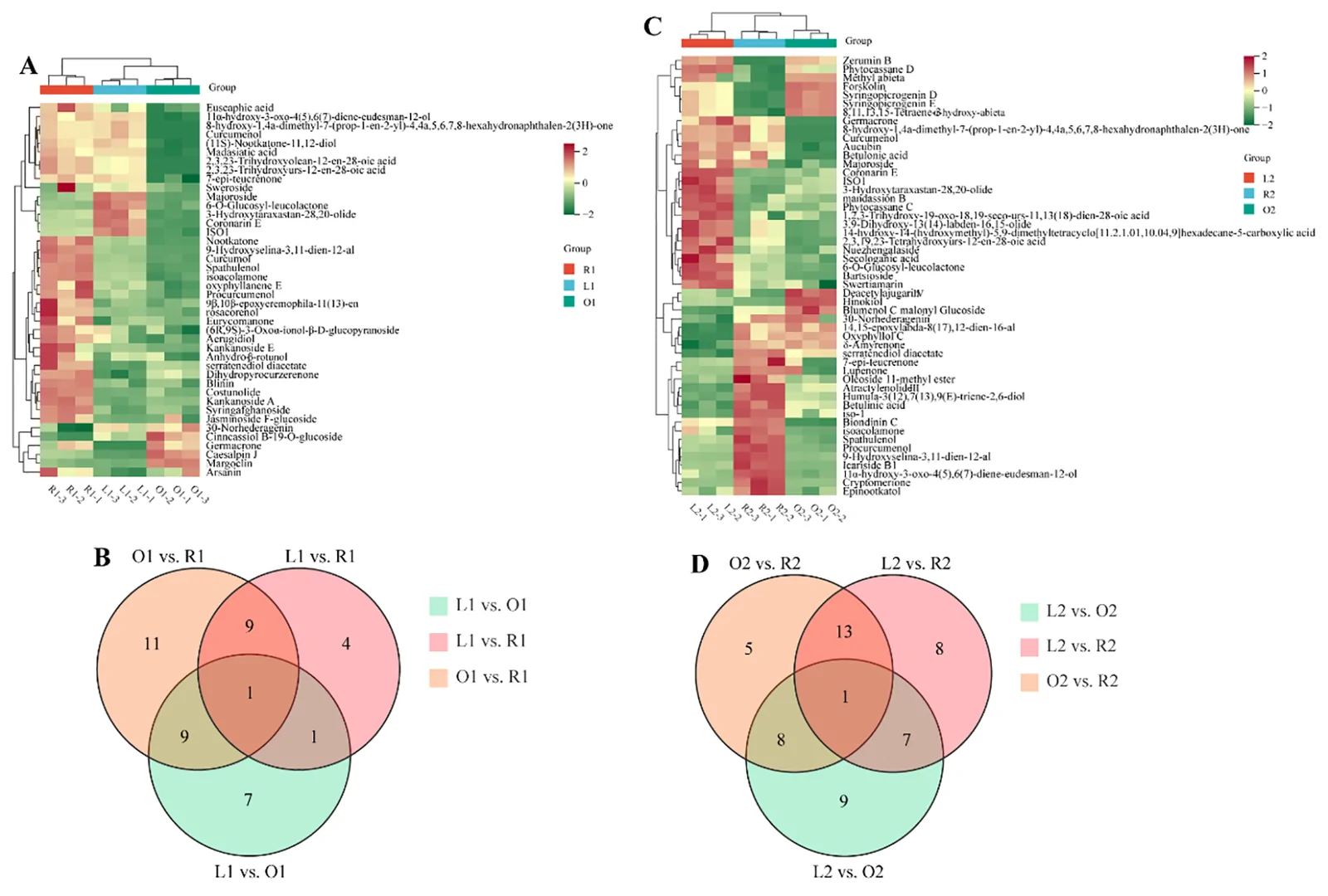
Comprehensive analysis of differential non-volatile terpenoid metabolites in *Amomum tsao-ko* fruits of different shapes. **(A)** Heatmap of differential non-volatile terpenoid metabolites in fresh fruits of different shapes. **(B)** Heatmap of differential non-volatile terpenoid metabolites in dried fruits of different shapes. **(C)** Venn diagram of differential non-volatile terpenoid metabolites for each comparison group in fresh fruits of different shapes. **(D)** Venn diagram of differential non-volatile terpenoid metabolites for each comparison group in dried fruits of different shapes. In the heatmaps, the color scale from red to green represents relative metabolite abundance from high to low (high = red, low = green). Hierarchical clustering was performed using Euclidean distance as the distance metric and complete linkage as the agglomeration method.

A total of 42 unique differential non-volatile terpenoid metabolites (DNVTMs) were identified across the three fresh fruit comparison groups (L1 vs. O1, L1 vs. R1, O1 vs. R1), whose accumulation patterns are visualized in the heatmap (**Figure 4A**). Pairwise comparisons revealed distinct metabolic profiles: the L1 vs. O1, L1 vs. R1, and O1 vs. R1 groups contained 18, 15, and 30 DNVTMs, respectively (**Figure 4B**). The O1 vs. R1 comparison showed the most pronounced differences, with 26 metabolites up-accumulated and 4 down-accumulated in R1 relative to O1.

Specifically, compared to long-shaped fresh fruits (L1), oval-shaped fresh fruits (O1) exhibited down-accumulation of 15 non-volatile terpenoids, including 4 monoterpenoids, 5 sesquiterpenoids, 1 diterpenoid, and 5 triterpenoids. In contrast, 3 non-volatile terpenoids were up-accumulated in O1: one monoterpenoid (Margoclin) and two sesquiterpenoids (Caesalpin J and Germacrone). Notably, Germacrone was undetectable in L1, while Curcumenol and 2,3,23-Trihydroxyolean-12-en-28-oic acid were absent in O1. In the comparison between L1 and round-shaped fresh fruits (R1), 13 terpenoids (3 monoterpenoids, 8 sesquiterpenoids, 1 diterpenoid, and 1 triterpenoid) were up-accumulated in R1, whereas 2 metabolites (1 monoterpenoid and 1 sesquiterpenoid) were down-accumulated.

Overall, the majority of differential terpenoids followed a content order of round-shaped fresh fruits > long-shaped fresh fruits > oval-shaped fresh fruits. Venn analysis identified Caesalpin J, a sesquiterpenoid, as the sole common DNVTMs across all three comparison groups. Its content consistently followed the order O1 > R1 > L1, highlighting its potential as a reliable marker for discriminating fresh fruit shapes.

#### 3.3.2 Differential non-volatile terpenoids among dried fruits of different shapes

Analysis of dried fruit samples further revealed the significant impact of fruit shape on non-volatile terpenoid metabolites. After deduplication, a total of 51 DNVTMs were identified across the three dried fruit comparison groups (L2 vs. O2, L2 vs. R2, O2 vs. R2) (**Figure 4C**), whose accumulation patterns are clearly displayed in the heatmap. The number of differential metabolites (L2 vs. O2: 25, L2 vs. R2: 29, O2 vs. R2: 27) was significantly higher than in the corresponding fresh fruit groups (**Figure 4D**), suggesting that the drying process may amplify or highlight metabolic differences between shapes. These differential metabolites comprised 10 monoterpenoids, 18 sesquiterpenoids, 12 diterpenoids, 1 terpene and 10 triterpenoids.

Specifically, in the comparison between long-shaped (L2) and oval-shaped (O2) dried fruits, the vast majority of DNVTMs (19/25) were down-accumulated in O2. This included sesquiterpenoids like Germacrone and Curcumenol, as well as several monoterpenoids (e.g., 6-O-Glucosyl-leucolactone, Majoroside). Notably, Germacrone and Curcumenol were undetectable in O2. In contrast, a few metabolites such as the diterpenoid Hinokiol and the sesquiterpenoid Blumenol C malonyl Glucoside were significantly up-accumulated in O2.

The trend shifted markedly in the comparison between long-shaped (L2) and round-shaped (R2) dried fruits. Among the 29 DNVTMs, 17 were up-accumulated in R2. This was particularly evident for sesquiterpenoids; for instance, the content of 9-Hydroxyselina-3,11-dien-12-al in R2 was approximately 13-fold higher than in L2, and Procurcumenol and Humula-3(12),7(13),9(E)-triene-2,6-diol also increased significantly. However, some metabolites, including the sesquiterpenoid mandassion B, the monoterpenoid Syringopicrogenin D, and several diterpenoids (e.g., Coronarin E, Forskolin), were down-accumulated in R2.

The comparison between oval-shaped (O2) and round-shaped (R2) dried fruits showed that most DNVTMs (17/27) were present at higher levels in R2. This was especially true for Sesquiterpenoids like Cryptomerione, Germacrone, and Curcumenol, which were notably up-accumulated in R2. An exception was the pair of monoterpenoids, Syringopicrogenin D/E, whose content in O2 was 5.7 times that in R2. Venn analysis identified the triterpenoid 3-Hydroxytaraxastan-28,20-olide as the common differential metabolite across all dried fruit comparison groups.

Comprehensive analysis indicates that post-drying, the terpenoid metabolic profiles of *A. tsao-ko* fruits with different shapes become more complex and distinct. Unlike the general round > long > oval content trend observed in fresh fruits, the accumulation patterns of metabolites in dried fruits varied depending on the compound class and specific comparison group. For example, round-shaped dried fruits showed an advantage in multiple sesquiterpenoids. These findings suggest that fruit shape not only influences the metabolic foundation of fresh fruits but is also closely related to the final chemical composition after drying and processing, which may subsequently affect the material basis of its efficacy or quality characteristics.

#### 3.3.3 Analysis of differential non-volatile terpenoid metabolites between fresh and dried *Amomum tsao-ko* fruits

**Figure 5:**
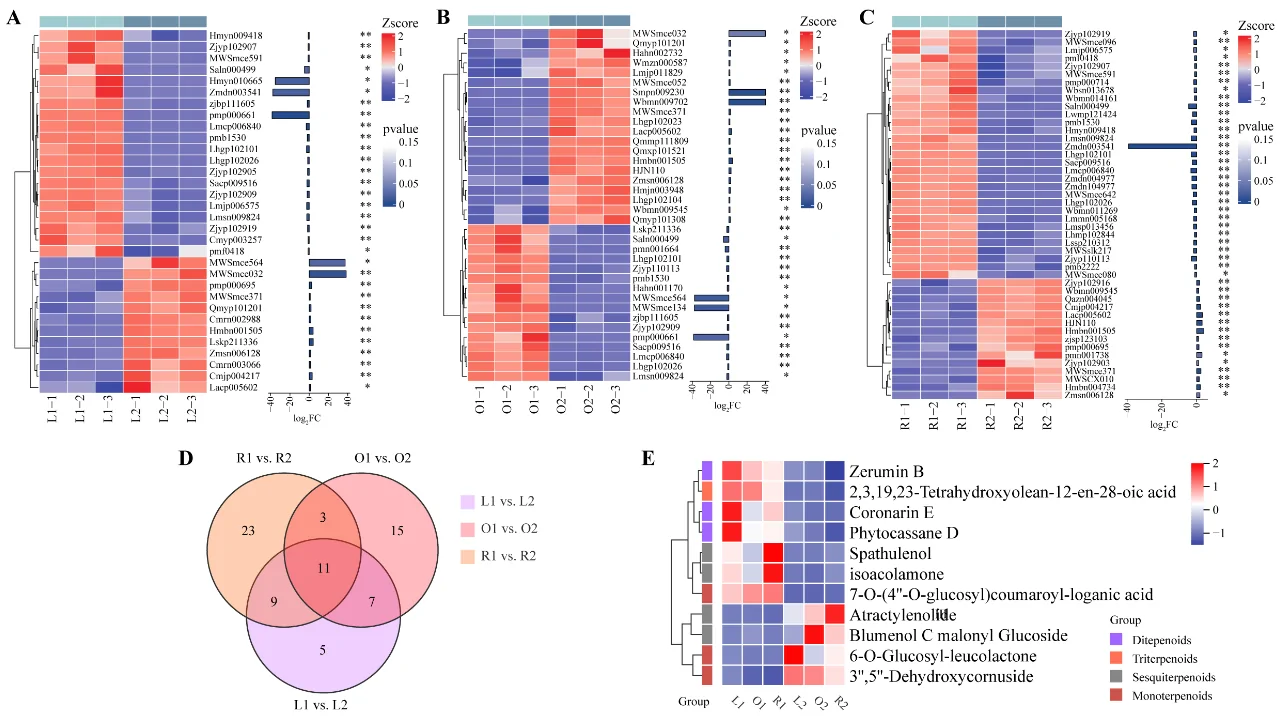
Comprehensive analysis of differential non-volatile terpenoid metabolites between fresh and dried *Amomum tsao-ko* fruits. **(A)** Heatmap of differential non-volatile terpenoid metabolites between fresh and dried long-shaped fruits. **(B)** Heatmap of differential non-volatile terpenoid metabolites between fresh and dried oval-shaped fruits. **(C)** Heatmap of differential non-volatile terpenoid metabolites between fresh and dried round-shaped fruits. **(D)** Venn diagram of differential non-volatile terpenoid metabolites for each comparison group between fresh and dried fruits. **(E)** Heatmap of the 11 common differential non-volatile terpenoid metabolites shared by all comparison groups between fresh and dried fruits. In the heatmaps, the color scale from red to blue represents relative metabolite abundance from high to low (high = red, low = blue). Hierarchical clustering was performed using Euclidean distance as the distance metric and complete linkage as the agglomeration method.

Drying induced substantial and shape-dependent alterations in the non-volatile terpenoid profile of *A. tsao-ko* fruits. Pairwise comparisons between fresh and dried fruits for each shape identified 32, 36, and 46 DNVTMs for long (L1 vs. L2), oval (O1 vs. O2), and round (R1 vs. R2) fruits, respectively (**Figures 5A–C**), highlighting that the metabolic impact of drying is most pronounced in round-shaped fruits.

Across all shapes, the drying process led to a predominant downregulation of sesquiterpenoids and diterpenoids. In long-shaped fruits (L1 vs. L2), 20 metabolites were down-accumulated post-drying, including key sesquiterpenoids like Spathulenol (log_2_FC = –3.04) and isoacolamone (log_2_FC = –2.36), and diterpenoids such as Coronarin E (log_2_FC = –2.75) and Zerumin B (log_2_FC = –1.62). Notably, Cryptomerione and Syringafghanoside, undetectable in L2, accumulated significantly in L1. Conversely, several monoterpenoid glycosides, including 6-O-Glucosyl-leucolactone (log_2_FC = 4.10) and Caesalpin J (log_2_FC = 4.18), were dramatically up-accumulated.

A similar but distinct pattern was observed in oval-shaped fruits (O1 vs. O2), where 16 metabolites were down-accumulated and 20 up-accumulated. While sesquiterpenoids like Spathulenol and isoacolamone were again down-accumulated, the diterpenoid Hinokiol exhibited a marked 4-fold increase (log_2_FC = 2.01) in O2. The monoterpenoid 6-O-Glucosyl-leucolactone also showed significant up-accumulation (log_2_FC = 3.87).

The round-shaped fruits (R1 vs. R2) exhibited the most extensive changes, with 31 metabolites down-accumulated. This group showed severe depletion of multiple sesquiterpenoids (e.g., Spathulenol, log_2_FC = –3.31; isoacolamone, log_2_FC = –2.52) and diterpenoids. However, consistent with other shapes, monoterpenoids like 6-O-Glucosyl-leucolactone (log_2_FC = 4.18) and the sesquiterpenoid Atractylenolide III (log_2_FC = 2.47) were strongly up-accumulated upon drying.

Venn analysis of these three comparison groups revealed a core set of 11 common DNVTMs (**Figure 5D**), comprising 3 monoterpenoids, 4 sesquiterpenoids, 3 diterpenoids, and 1 triterpenoid. This identifies a conserved drying-responsive terpenoid pathway in *A. tsao-ko*, irrespective of fruit shape. Clustering heatmap analysis of these common metabolites clearly segregated fresh and dried samples (**Figure 5E**). Four metabolites, including Atractylenolide III (sesquiterpenoid), Blumenol C malonyl Glucoside (sesquiterpenoid), 6-O-Glucosyl-leucolactone (monoterpenoid), and 3’’,5’’-Dehydroxycornuside (monoterpenoid) were consistently up-accumulated in all dried fruits. In stark contrast, seven metabolites, including Spathulenol, isoacolamone, 7-O-(4’’-O-glucosyl)coumaroyl-loganic acid, Coronarin E, Phytocassane D, Zerumin B, and 2,3,19,23-Tetrahydroxyolean-12-en-28-oic acid, were uniformly down-accumulated after drying.

In summary, drying profoundly reshapes the non-volatile terpenoid metabolome of *A. tsao-ko*. The process consistently promotes the accumulation of specific monoterpenoid and sesquiterpenoid glycosides while depleting a range of free sesquiterpenoids and diterpenoids. The extent of these changes is modulated by fruit shape, with round fruits experiencing the most dramatic metabolic reprogramming. The identification of a conserved set of drying-responsive metabolites provides key chemical markers for understanding post-harvest processing effects on *A. tsao-ko* quality. This pattern suggests that drying might either stimulate glycosylation processes or preferentially degrade/dehydrate the aglycone forms of these terpenoids.

### 3.4 Transcriptomic basis of shape-dependent terpenoid variation

**Figure 6:**
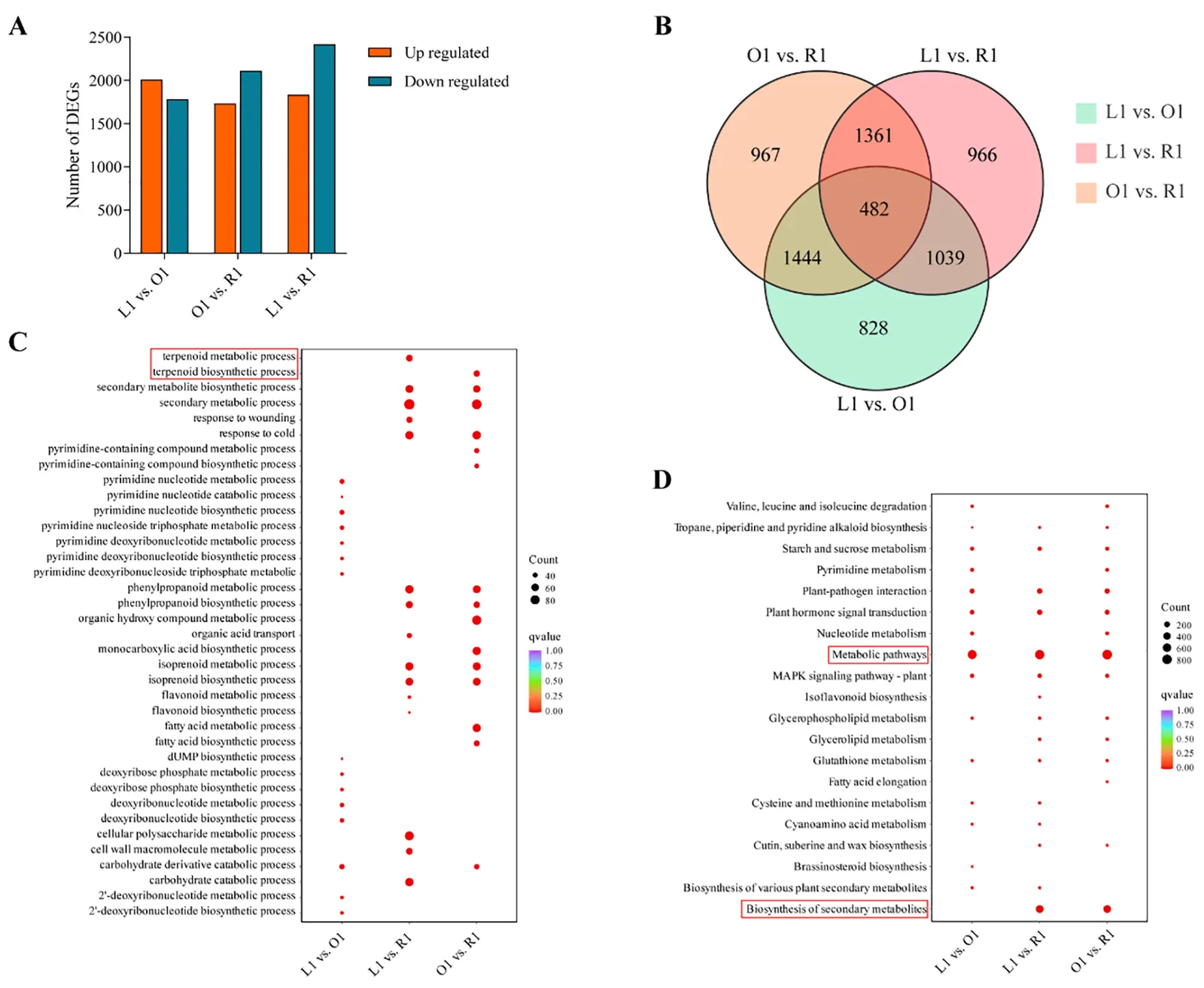
Transcriptomic data analysis of fresh *Amomum tsao-ko* fruits with different shapes. **(A)** Statistics of differentially expressed genes (DEGs) in different fresh fruit comparison groups. **(B)** Venn diagram of DEGs across different fresh fruit comparison groups. **(C)** GO functional annotation of common DEGs shared by the three comparison groups. **(D)** KEGG functional annotation of common DEGs shared by the three comparison groups.

To elucidate the transcriptional mechanisms underlying the distinct terpenoid accumulation patterns among different fruit shapes, RNA-seq analysis on the fresh fruits (L1, O1, R1) were conducted. A total of 28645 expressed genes were detected. Pairwise comparisons revealed substantial transcriptomic reprogramming: 3793, 3848, and 4254 DEGs were identified in L1 vs. O1, L1 vs. R1, and O1 vs. R1, respectively (**Figure 6A**; **Supplementary Table 3**). Venn analysis identified 482 DEGs common to all three comparisons (**Figure 6B**), representing a core set of shape-responsive transcripts.

Functional annotation of these common DEGs provided direct links to the observed metabolic phenotypes. Gene Ontology (GO) analysis highlighted significant enrichment in terms directly related to terpenoid metabolism, including terpenoid biosynthetic process (GO:0016114, 49 DEGs) and terpenoid metabolic process (GO:0006721, 52 DEGs) (**Figure 6C**). Kyoto Encyclopedia of Genes and Genomes (KEGG) pathway enrichment further confirmed that these core DEGs were predominantly concentrated in metabolic pathways (ko01100) and biosynthesis of secondary metabolites (ko01110) (**Figure 6D**). These results conclusively demonstrate that genetic regulation at the transcriptional level is a key driver of the shape-specific terpenoid profiles in *A. tsao-ko* fruits.

### 3.5 Identification of terpenoid-associated gene co-expression modules via WGCNA

**Figure 7:**
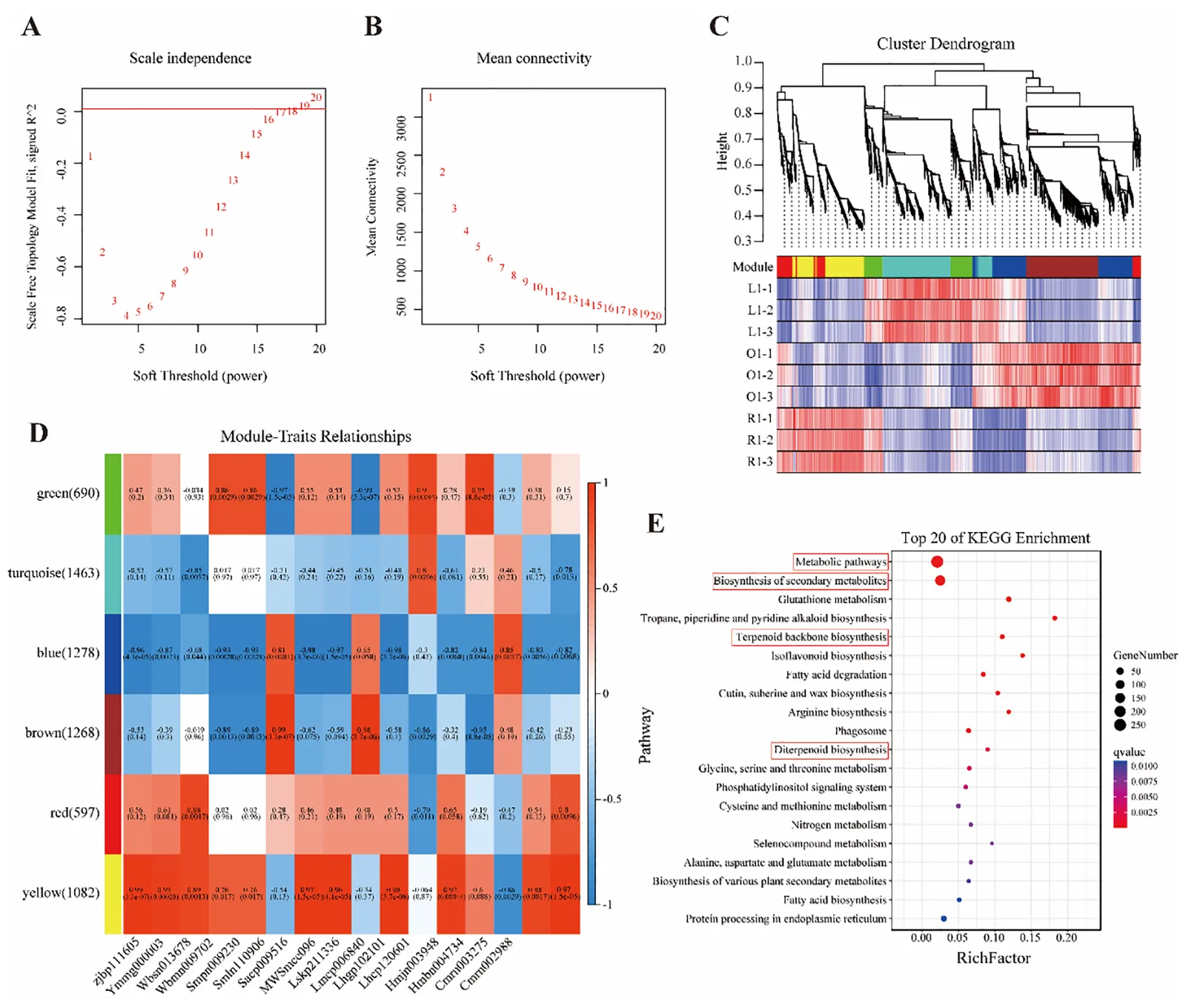
Weighted gene co-expression network analysis (WGCNA) Based on key non-volatile terpenoid metabolites. **(A)** Scale independence. **(B)** Mean connectivity. **(C)** Module identification. **(D)** Correlation analysis between modules and key non-volatile terpenoid metabolites. **(E)** KEGG enrichment analysis of genes in the yellow module.

To systemically identify gene modules co-expressed with the accumulation of key non-volatile terpenoids, we performed Weighted Gene Co-expression Network Analysis (WGCNA) using the transcriptome data from all fresh fruit samples. Based on the scale independence and average connectivity (**Figures 7A, B**), a soft-thresholding power of 18 was selected to construct a signed network. This analysis partitioned the expressed genes into six distinct co-expression modules, each labeled with a unique color (**Figure 7C**).

Module-trait correlation analysis, which assessed the relationship between each module’s eigengene and the abundance of critical terpenoid metabolites, identified the yellow module as the most significant (**Figure 7D**). This module, comprising 1,082 genes, showed the strongest positive correlations with the content of 11 key terpenoids, including monoterpenoids (Kankanoside E, Kankanoside A), sesquiterpenoids (Spathulenol, rosacorenol, isoacolamone, 9-Hydroxyselina-3,11-dien-12-al, 9β,10β-epoxyeremophila-11(13)-en, Nootkatone), and triterpenoids (2,3,23-Trihydroxyolean-12-en-28-oic acid, 2,3,23-Trihydroxyurs-12-en-28-oic acid, serratenediol diacetate). Conversely, it exhibited a significant negative correlation with the triterpenoid 30-Norhederagenin (Hmbn004734). KEGG enrichment analysis of genes within the yellow module confirmed its central role in terpenoid metabolism, showing significant enrichment in terpenoid backbone biosynthesis and diterpenoid biosynthesis pathways (**Figure 7E**). Thus, the yellow module was pinpointed as a key transcriptional network associated with terpenoid accumulation.

### 3.6 Characterization of key genes and regulatory network within the terpenoid-associated module

**Figure 8:**
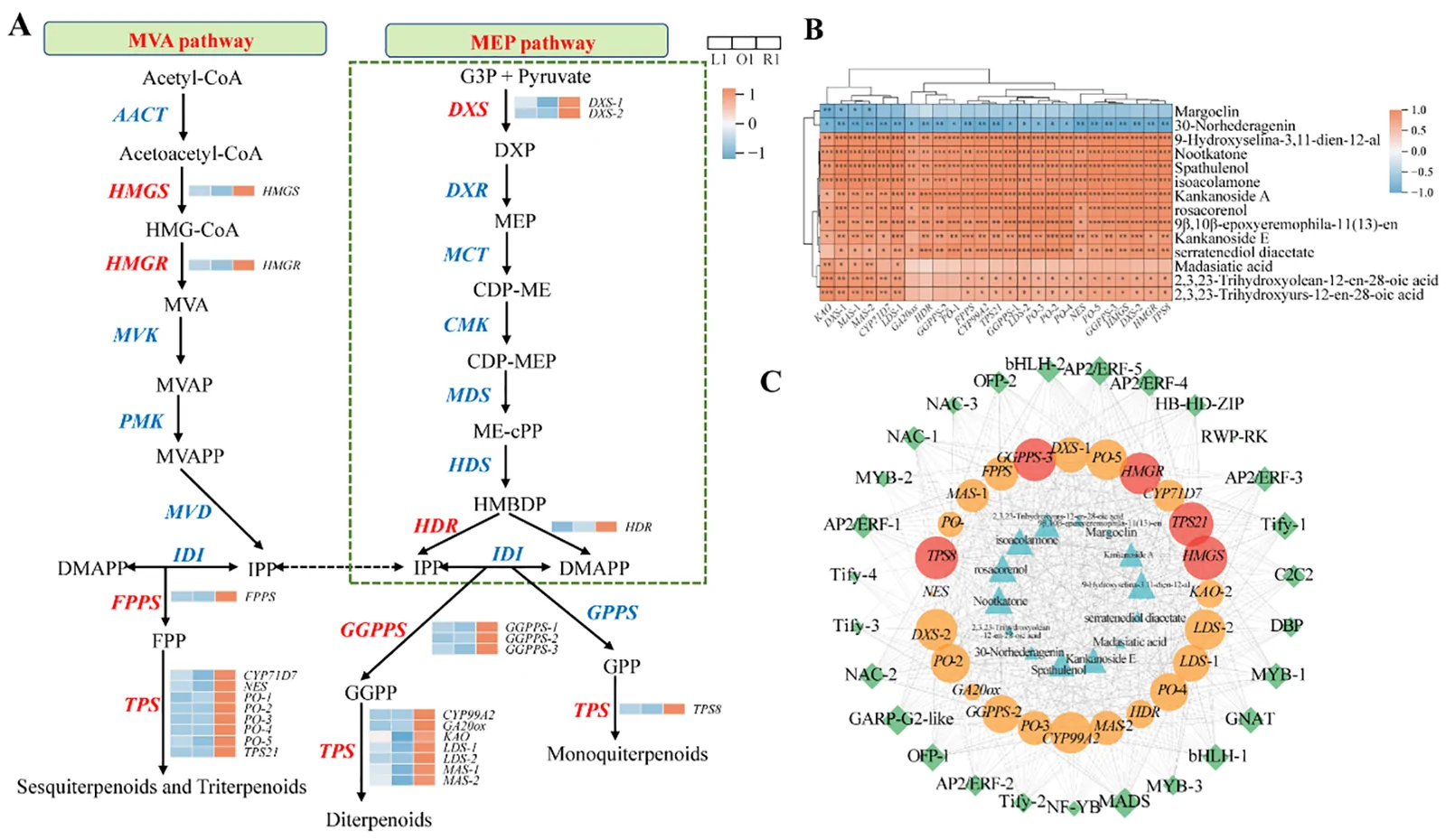
Metabolic pathway analysis of non-volatile terpenoids. **(A)** MVA and MEP metabolic pathways. **(B)** Correlation analysis between key non-volatile terpenoid metabolites and key candidate genes. **(C)** Co-expression analysis among key non-volatile terpenoid metabolites, key candidate genes, and key transcription factors.

A particular focus of this study was to identify key genes involved in terpenoid biosynthesis. Therefore, candidate genes responsible for terpenoid biosynthesis in the yellow module were further examined. As a result, multiple genes encoding enzymes related to terpenoid biosynthesis were identified in the yellow module. These genes in the yellow module covered the entire terpenoid synthesis pathway (**Figure 8A**), 6 terpenoid backbone genes: Including 2 genes encoding 1-deoxy-D-xylulose-5-phosphate synthase (DXS), 1 gene encoding farnesyl diphosphate synthase (FPPS), 3 genes encoding geranylgeranyl diphosphate synthase (GGPPS), 1 gene encoding hydroxymethylglutaryl-CoA reductase (HMGR), 1 gene encoding hydroxymethylglutaryl-CoA synthase (HMGS), and 1 gene encoding 4-hydroxy-3-methylbut-2-en-1-yl diphosphate reductase (HDR); 5 diterpenoid biosynthesis pathway genes: Including 1 gene encoding 9beta-pimara-7,15-diene oxidase, 1 gene encoding gibberellin 20 oxidase (GA20ox), 1 gene encoding ent-kaurenoic acid monooxygenase (KAO), 2 genes encoding (13E)-labda-7,13-dien-15-ol synthase (LDS), and 2 genes encoding momilactone-A synthase (MAS); 8 sesquiterpene synthase genes: Including 1 gene encoding 5-epiaristolochene 1,3-dihydroxylase, 1 gene encoding (3S,6E)-nerolidol synthase (NES), 5 genes encoding premnaspirodiene oxygenase (PO), and 1 gene encoding alpha-humulene/beta-caryophyllene synthase (TPS21); 1 monoterpene synthase gene: Encoding (-)-alpha-terpineol synthase (TPS8).

The expression patterns of these candidate biosynthetic genes within the yellow module showed significant correlations with the accumulation of the aforementioned key terpenoids (**Figure 8B**). For instance, several GGPPS and LDS genes exhibited strong positive correlations with diterpenoid precursors and downstream metabolites. Similarly, sesquiterpene synthase genes like TPS21 and PO showed co-expression patterns with specific sesquiterpenoids such as Spathulenol and Nootkatone. This correlation network directly links the transcriptional activity of specific pathway genes to the quantitative variation of definitive terpenoid products.

Furthermore, we identified 27 transcription factors (TFs) within the yellow module, belonging to families such as *AP2*/*ERF*, *bHLH*, *MYB*, *NAC*, and *MADS*. Co-expression network analysis (**Figure 8C**) demonstrated that the expression of these TFs was highly correlated (Pearson correlation coefficient > 0.80) with the structural genes described above. Notably, *AP2*/*ERF-4*, *AP2*/*ERF-5*, *GARP-G2*-like, *MADS*, *OFP-1*, and *Tify-1* were co-expressed with multiple structural genes across the terpenoid pathway. This suggests that these TFs are potential key regulators orchestrating the coordinated expression of biosynthetic genes, thereby modulating the synthesis and accumulation of non-volatile terpenoids in *A. tsao-ko* fruits in a shape-dependent manner.

## 4 Discussion

### 4.1 Richness and conservation of terpenoids in *A. tsao-ko*

This study provides the first comprehensive profiling of non-volatile terpenoids in the fruits of *A. tsao-ko*, identifying 157 metabolites spanning monoterpenoids, sesquiterpenoids, diterpenoids, triterpenoids, and other terpenes. The high proportion of sesquiterpenoids (38.85%) aligns with previous reports in other Zingiberaceae species, such as *Curcuma longa* and *Zingiber officinale*, where sesquiterpenoids dominate the terpenoid profile and contribute significantly to bioactivity (Zhang et al., 2023). This suggests a conserved metabolic emphasis on sesquiterpenoid biosynthesis within the family, possibly linked to their roles in chemical defense and environmental adaptation (Pichersky and Raguso, 2018). The remarkable overlap of 147 terpenoids (93.63%) across all samples, regardless of shape or processing state, underscores a stable core metabolome in *A. tsao-ko*. Such conservation likely reflects fundamental physiological and ecological functions, including pathogen resistance and herbivore deterrence, which are maintained across morphotypes (Pichersky and Raguso, 2018).

Several terpenoids identified here have known pharmacological relevance. Isosteviol, detected exclusively in dried fruits, is a steviol derivative with documented antibacterial, antitumor, and antihyperglycemic properties (Yang et al., 2024). Its formation or accumulation upon drying may result from dehydration-induced chemical conversion or stability under low-moisture conditions, offering a potential chemical marker for processed *A. tsao-ko*. The five consistently high-abundance terpenoids, including Ajugalaevigatic acid, Kobusone, Confertifoline, 6β-Hydroxy-8α-methoxyeremophila-1(10),7(11)-dien-12,8β-olide, and 3,9-Dihydroxy-13(14)-labden-16,15-olide constitute a robust chemical signature for this species. Ajugalaevigatic acid, a diterpenoid, exhibits cytotoxicity against cancer cell lines (Topcu et al., 2004), Kobusone stimulates pancreatic β-cell replication (Choi et al., 2021), Confertifoline shows antimicrobial and potential immunomodulatory activities (Duraipandiyan et al., 2010; Xia et al., 2021), and 3,9-Dihydroxy-13(14)-labden-16,15-olide possesses anti-inflammatory properties (Sichaem et al., 2021; Lee et al., 2013; Ban et al., 2020). Interestingly, 7-O-(4’’-O-glucosyl)coumaroyl-loganic acid, an iridoid monoterpenoid previously reported mainly in Gentianaceae (Xuan et al., 2024), was here detected in *A. tsao-ko* for the first time, expanding the known chemotaxonomic range of this compound. The stable abundance of these core terpenoids supports their utility as quality-control markers for *A. tsao-ko* fruits, irrespective of morphological variation.

### 4.2 Fruit shape-dependent variation in terpenoid profiles

Our results demonstrate that fruit shape significantly influences non-volatile terpenoid accumulation, both in fresh and dried states. In fresh fruits, round-shaped samples generally exhibited higher terpenoid contents than long- and oval-shaped fruits, with Caesalpin J emerging as a shape-discriminatory marker. This variation may reflect underlying genetic or developmental differences that regulate terpenoid biosynthetic flux. Similar shape- or genotype-dependent metabolite variations have been reported in other medicinal plants, such as *Houttuynia cordata* (Zhang et al., 2025), *Ocimum* (Du et al., 2023) and *Panax* species, where morphotype often correlates with distinct chemotypes (Abaya et al., 2024; Cho et al., 2012). In dried fruits, shape-related differences became more pronounced, with round fruits showing elevated levels of several sesquiterpenoids (e.g., Cryptomerione, Germacrone, Curcumenol). This amplification after drying suggests that shape-related structural traits (e.g., pericarp thickness, surface-to-volume ratio) may modulate water loss rates and thus affect the stability or transformation of specific terpenoids during dehydration (Corona et al., 2020). The absence of Curcumenol in oval-shaped fruits, both fresh and dried, indicates a genetically determined deficiency in the biosynthetic steps leading to these sesquiterpenoids. Germacrone, a major bioactive component in Curcuma species, exhibits anti-inflammatory and anticancer activities (Guo et al., 2024); its absence in oval-shaped *A. tsao-ko* could imply differential medicinal potency among morphotypes. These findings highlight that fruit shape is not merely a morphological trait but a determinant of chemical composition, which should be considered in the grading and standardization of *A. tsao-ko* for medicinal or culinary uses.

### 4.3 Impact of drying on terpenoid metabolism and potential mechanisms

Drying induced profound and shape-dependent alterations in the terpenoid profile. The consistent down-accumulation of free sesquiterpenoids (e.g., Spathulenol, isoacolamone) and diterpenoids (e.g., Coronarin E, Zerumin B), coupled with the up-accumulation of glycosylated monoterpenoids (e.g., 6-O-Glucosyl-leucolactone) and certain sesquiterpenoid lactones (e.g., Atractylenolide III), suggests that drying may promote glycosylation or dehydration-driven cyclization reactions while volatilizing or degrading more labile aglycones. Similar drying-induced shifts toward glycosylated forms have been observed in other medicinal plants (Liu et al., 2023), where glycosides often accumulate as stable storage forms under desiccation stress (Moyankova et al., 2014). The core set of 11 drying-responsive terpenoids common to all shapes indicates conserved biochemical responses to dehydration, possibly mediated by stress-responsive enzymes like glycosyltransferases or oxidoreductases. Round-shaped fruits experienced the most extensive metabolic reprogramming upon drying, which may be attributed to their higher surface area facilitating faster water loss and more intense oxidative or enzymatic activity. The appearance of Isosteviol exclusively in dried samples further supports that drying can generate unique compounds through dehydration-driven rearrangements, as reported for steviol glycosides in *Stevia* leaves (Orellana-Paucar, 2023; Momtazi-Borojeni et al., 2017). These changes underscore that post-harvest processing not only preserves the material but also actively transforms its chemical identity, thereby influencing the final quality and bioactivity of *A. tsao-ko*.

### 4.4 Transcriptional regulation underlying shape- and drying-associated terpenoid variation

RNA-seq analysis revealed substantial transcriptomic differences among fresh fruits of different shapes, with a core set of 482 differentially expressed genes (DEGs) enriched in terpenoid biosynthesis pathways. This provides a molecular basis for the observed metabolic variation and confirms that shape-dependent terpenoid accumulation is regulated at the transcriptional level. Similar transcript-metabolite correlations have been documented in *Cannabis sativa* (Booth et al., 2017; Zager et al., 2019) and *Ginkgo biloba* (Zheng et al., 2024), where chemotype differences are linked to differential expression of terpene synthase genes. WGCNA identified the yellow module as a key co-expression network positively correlated with the accumulation of multiple monoterpenoids, sesquiterpenoids, and triterpenoids. Within this module, structural genes encoding *DXS*, *FPPS*, *GGPPS*, *HMGR*, and various terpene synthases showed coordinated expression patterns with specific metabolites, indicating tight pathway regulation. Notably, the identification of 27 transcription factors (TFs) from families such as *AP2/ERF*, *bHLH*, *MYB*, and *NAC* co-expressed with these structural genes suggests a hierarchical regulatory network controlling terpenoid diversity. *AP2/ERF* and *MYB* TFs are known regulators of terpenoid biosynthesis in many plants (Wang et al., 2022; Guan et al., 2025), their involvement here implies conserved regulatory mechanisms. The shape-specific expression of these TFs may drive the differential activation of terpenoid pathways, ultimately leading to distinct chemical profiles. These insights lay a foundation for future functional studies to validate candidate genes and engineer terpenoid production in *A. tsao-ko*.

### 4.5 Limitations and future perspectives

While this study provides a comprehensive metabolomic and transcriptomic characterization of non-volatile terpenoids in *A. tsao-ko*, several limitations should be acknowledged. While our RNA-seq and WGCNA analyses have identified a set of candidate structural genes and transcription factors potentially regulating shape-dependent terpenoid accumulation, we acknowledge that the lack of independent qRT-PCR validation represents a limitation of this study. The extended storage of our field-collected samples further compromised RNA integrity, precluding reliable qPCR analysis. Therefore, functional validation of these candidate genes, including heterologous expression, gene silencing (e.g., VIGS or CRISPR/Cas9), and qRT-PCR confirmation using freshly collected materials will be essential in future studies to definitively confirm their roles in terpenoid biosynthesis and to fully validate the regulatory network proposed here. The study examined fruits from a single geographic location and harvesting time, seasonal and environmental variations could influence terpenoid profiles and should be investigated in future work.

Looking forward, multi-omics integration (metabolomics, transcriptomics, proteomics) across different developmental stages and environments will deepen our understanding of the spatiotemporal regulation of terpenoid biosynthesis in *A. tsao-ko*. Moreover, *in-vitro* and *in-vivo* pharmacological assays on the identified characteristic terpenoids (e.g., Isosteviol, Caesalpin J, core high-abundance compounds) are essential to elucidate their health-relevant bioactivities and justify their use as quality markers. Finally, breeding or biotechnological approaches could be explored to enhance desirable terpenoids in specific morphotypes, ultimately improving the consistency and efficacy of *A. tsao-ko* as a medicinal and culinary resource.

## 5 Conclusion

This study provides the first comprehensive profiling of non-volatile terpenoids in *Amomum tsao-ko*, revealing a rich and conserved metabolome with significant variation influenced by fruit morphotype and post-harvest drying. We identified 157 terpenoids, with five core high-abundance compounds forming a stable chemical signature. Fruit shape, particularly round morphotype, was associated with higher terpenoid content and distinct metabolic profiles, both in fresh and dried states. The drying process consistently promoted the accumulation of glycosylated terpenoids while reducing free forms, with round fruits undergoing the most extensive metabolic reprogramming. Transcriptomic analysis uncovered a shape-responsive gene set enriched in terpenoid biosynthesis, and WGCNA identified a co-expression module tightly linked to terpenoid accumulation, highlighting key structural genes and potential regulatory transcription factors. These integrated findings offer valuable insights for the quality grading, germplasm selection, and processing optimization of *A. tsao-ko*, supporting its sustainable development as a dual-purpose medicinal and culinary resource. Future studies should focus on functional validation of candidate genes and pharmacological evaluation of key terpenoids to further elucidate their bioactivities and commercial potential.

## Data Availability

The original sequencing data generated in the study have been deposited into the National Center for Biotechnology Information (NCBI) Sequence Read Archive (SRA) database with the primary accession code PRJNA1072740.
