# Supplementary material for: Multi-omics analysis reveals shape- and drying-dependent variation in non-volatile terpenoids of Amomum tsao-ko fruits
Source: Front Plant Sci. 2026 Sep 3;17:1937993. doi: 10.3389/fpls.2026.1937993 (PMC13582298; doi:10.3389/fpls.2026.1937993)
Supplement: Supplementary file 1 [file DataSheet1.docx]

Figure S1. Length-to-width ratio (LWR) of different fruit shapes in *A. tsao-ko*. One-way ANOVA followed by Duncan's multiple range test at the 0.05 significance level.
